# Public Perceptions and Influencing Factors of Non-National Immunization Program (Non-NIP) Vaccines in Shanghai: A Population-Based Study

**DOI:** 10.3390/vaccines14020174

**Published:** 2026-02-13

**Authors:** Haifeng Ma, Yu Zhang, Danni Zhao, Hongmei Lu, Ping Yu, Jialei Fan, Qiangsong Wu, Wenjiang Zhong, Huiyong Shao, Xiaodong Sun, Zhuoying Huang, Linlin Wu

**Affiliations:** 1 Shanghai Municipal Center for Disease Control & Prevention, Shanghai 200336, China; mahaifeng@scdc.sh.cn (H.M.); sunxiaodong@scdc.sh.cn (X.S.); 2 Shanghai Songjiang District Municipal Center for Disease Control & Prevention, Shanghai 201620, China; 3 Shanghai Jingan District Municipal Center for Disease Control & Prevention, Shanghai 200072, China; 4 Shanghai Minhang District Municipal Center for Disease Control & Prevention, Shanghai 201101, China; 5 Shanghai Xuhui District Municipal Center for Disease Control & Prevention, Shanghai 200237, China; 6 Shanghai Qingpu District Municipal Center for Disease Control & Prevention, Shanghai 201799, China

**Keywords:** non-NIP vaccine, awareness level, influencing factors, acceptance level

## Abstract

This study aimed to explore the cognitive levels and influencing factors of Shanghai residents regarding non-immunization program vaccines. A population-based study was conducted in Shanghai in 2024. **Objective:** To examine awareness levels and factors influencing perceptions of non-National Immunization Program (non-NIP) vaccines among residents of Shanghai. **Methods:** A population cross-sectional survey was conducted in Shanghai from 20 October to 31 December 2024, using stratified random sampling. Five districts were selected, four communities per district were randomly chosen, and 35–40 residents per community were invited to complete a questionnaire. Data collected included sociodemographic characteristics, awareness of non-NIP vaccines, and potential influencing factors. Awareness and acceptance of non-NIP vaccines were measured using five-point Likert scales. On a 0–4 scale, where 0 = completely unaware/unsupportive and 4 = very aware/strongly supportive, respondents rated their level of understanding and endorsement of non-NIP vaccines. Descriptive analysis, the Kruskal–Wallis test, and ordinal logistic regression were used to assess awareness levels and their determinants. **Results:** Among the 753 respondents, 15.5% of respondents reported very high awareness, 18.7% reported fairly high awareness, 32.1% reported moderate awareness, 27.2% reported somewhat low awareness, and 6.5% reported complete unawareness. Acceptance levels were distributed as follows: 20.3% strongly in favour, 24.7% somewhat in favour, 45.9% neutral, 7.3% somewhat opposed, and 1.9% strongly opposed. Higher awareness was significantly associated with younger age, higher household living standard, receiving a recommendation from medical personnel, and participation in vaccine education programs (all *p* < 0.05). Acceptance was significantly influenced by age, residence type (urban community, town center, or rural), medical personnel recommendation, educational campaign participation, and perceived affordability of vaccine cost (all *p* < 0.05). **Conclusions:** Overall, Shanghai residents exhibited suboptimal awareness and acceptance of non-NIP vaccines, with a clear “high acceptance but low knowledge” phenomenon. To improve awareness, strategies should include strengthening healthcare providers’ recommendations and implementing systematic educational campaigns. To enhance acceptance, efforts should focus on disseminating positive, evidence-based information; reinforcing provider guidance; expanding outreach and education; and optimizing payment mechanisms to improve economic accessibility.

## 1. Introduction

Vaccination is a cornerstone of public health, playing a critical role in preventing and controlling infectious diseases [1]. In China, vaccines are broadly categorized into National Immunization Program (NIP) vaccines and non-National Immunization Program (non-NIP) vaccines. NIP vaccines are funded and provided free of charge by the government as part of routine public health services and are administered according to the national immunization schedule, such as the hepatitis B vaccine, Bacillus Calmette–Guérin (BCG) vaccine, inactivated poliovirus vaccine (IPV), attenuated oral poliovirus vaccine (OPV), diphtheria–tetanus–pertussis (DTP) vaccine, measles–mumps–rubella (MMR) vaccine, live and inactivated Japanese encephalitis vaccines, group A and group A/C meningococcal polysaccharide vaccines, and hepatitis A vaccines (both live and inactivated). The routine vaccination coverage rate for NIP vaccines among eligible children in China has been consistently maintained above 90%, reflecting long-standing success in preventing major childhood infectious diseases.

Non-National Immunization Program vaccines refer to vaccines not included in China’s National Immunization Program and administered voluntarily at individuals’ expense [2]. Common non-NIP vaccines in China include rabies vaccine, seasonal influenza vaccine, varicella (chickenpox) vaccine, rotavirus vaccine, enterovirus 71 (EV71) vaccine, and pneumococcal conjugate vaccines (PCVs), among others. Among non-NIP vaccines, rabies, influenza, and varicella vaccines are typically the most frequently administered in China. Existing evidence from clinical trials and real-world studies has demonstrated that these vaccines are effective in reducing disease incidence and related complications across different age groups [3,4,5,6]. Compared with vaccines included in China’s National Immunization Program (NIP), vaccination coverage for non-NIP vaccines was substantially lower. Relevant research indicated that the full primary series coverage for 13-valent pneumococcal conjugate vaccine (PCV13) was approximately 5.1% in 2019, rotavirus vaccine three-dose coverage was about 1.8%, and Haemophilus influenzae type b (Hib) vaccine three-dose coverage was approximately 25.0%; only first-dose varicella vaccine approached higher levels (around 67.1%) in selected settings, reflecting localized inclusion in some municipal programs rather than national free provision. These figures illustrated that non-NIP vaccine uptake remained far below the near-universal coverage of NIP vaccines and exhibited substantial regional and product-specific disparities [7].

A growing body of economic evaluation studies in China suggests that several non-NIP vaccines, particularly PCV, HPV, Hib, and influenza vaccines, demonstrate favorable cost-effectiveness profiles compared with standard thresholds based on GDP per capita, supporting their potential public health value in appropriate populations [8,9]. Different non-National Immunization Program (non-NIP) vaccines are indicated for distinct population groups based on disease risk and prevention objectives. For example, pneumococcal conjugate vaccines (PCVs) and Haemophilus influenzae type b (Hib) vaccines are primarily used in infants and young children to prevent invasive bacterial infections such as pneumonia and meningitis, rotavirus vaccines are administered to young children to prevent severe diarrhoeal disease, human papillomavirus (HPV) vaccines target adolescents and young adults to prevent HPV-associated cancers, and seasonal influenza vaccines are recommended for children, older adults, pregnant women, and other high-risk groups to reduce influenza-related morbidity and mortality. Some non-National Immunization Program (non-NIP) vaccines in China are regarded as alternative vaccines because they can replace vaccines already included in the National Immunization Program (NIP) by using formulations with broader antigenic coverage or combined components. For example, the pentavalent combination vaccine (DTaP-IPV-Hib) integrates diphtheria, tetanus, acellular pertussis, inactivated poliovirus, and Haemophilus influenzae type b antigens into a single product, thereby substituting for multiple separate NIP vaccines while reducing the number of injections. Similarly, quadrivalent meningococcal conjugate vaccines (ACWY) provide broader serogroup protection than monovalent group A meningococcal vaccines, and nine-valent human papillomavirus (HPV) vaccines extend protection beyond bivalent formulations by covering additional oncogenic HPV types.

The extent to which these alternative non-NIP vaccines are used varies across regions and vaccine types, depending on local supply, service practices, and population demand. These alternative non-NIP vaccines are typically self-paid because they are not included in the publicly funded NIP schedule. From the perspective of vaccine recipients, the primary motivations for choosing these self-paid alternatives are their favorable safety profiles and the reduced number of injections achieved through combination vaccines.

As highly cost-effective interventions, vaccines significantly reduce disease burden and safeguard population health. non-NIP vaccines serve as important supplements or alternatives to NIP vaccines in controlling infections and meeting diverse immunization needs [10]. However, vaccination coverage for non-NIP vaccines remains low in China, with notable regional disparities [11,12]. These gaps may be partly attributable to limited public awareness and acceptance of such vaccines, as well as socioeconomic and informational factors [13,14,15,16,17].

In recent years, an increasing body of research has underscored the importance of understanding public knowledge and attitudes toward non-National Immunization Program (non-NIP) vaccines. Evidence indicates that the general population’s level of awareness and their attitudes toward non-NIP vaccines are significantly associated with their vaccination behaviors and uptake rates [18,19]. In particular, studies across different regions have identified low awareness and misconceptions regarding the safety, efficacy, or necessity of non-NIP vaccines as major barriers hindering their wider adoption [20].

Recent introduction of new vaccines has exerted a dual impact on public vaccine acceptance: initially fostering widespread willingness to vaccinate, but over time leading to divergence in attitudes due to misinformation dissemination and erosion of institutional trust—thereby challenging the sustained effectiveness of immunization strategies [21]. Empirical work indicates that individuals’ expectations and mental imagery regarding potential disease risk and possible vaccine adverse effects jointly contribute to general vaccine hesitancy. For example, previous research showed that among unvaccinated individuals, more vivid negative imagery about disease risk combined with less vivid negative imagery about vaccine side effects predicted lower hesitancy and higher vaccine uptake; whereas among vaccinated individuals, it was the interaction between risk expectations (rather than mental imagery) that predicted hesitancy [22]. Moreover, studies have documented that public vaccine sentiment exhibited a clear inverted-U shaped trajectory over time in relation to periods of intensive vaccine rollout and media coverage [23,24].

Empirical studies across diverse settings have consistently demonstrated that population-level vaccine-related knowledge and attitudes exert a substantial influence on vaccination uptake [25,26]. Multiple studies have demonstrated that higher vaccine knowledge and positive attitudes are strongly associated with greater vaccine uptake and acceptance, highlighting the importance of cognitive and belief factors in vaccination behavior [27,28]. Conversely, widespread misconceptions and negative perceptions about vaccines can significantly reduce vaccination rates by increasing hesitancy and undermining public confidence [29,30,31].

In the Chinese context, empirical studies have identified several determinants of vaccine awareness and acceptance. For example, a survey of the general adult population in China, found that knowledge and positive attitudes toward vaccine efficacy and safety were significantly associated with willingness to vaccinate, suggesting that higher levels of disease and vaccine knowledge can enhance acceptance in large-scale immunization campaigns [32]. Moreover, systematic reviews of HPV vaccination in mainland China have shown that awareness of the vaccine, understanding of disease risk, perceived vaccine safety, and cost considerations are among the key predictors of willingness to accept non-NIP vaccines, indicating that both informational and socioeconomic factors play important roles in shaping vaccine decisions [33]. In Shanghai, immunization services are delivered through an integrated public health network coordinated by the Centers for Disease Control and Prevention and community health centers, where National Immunization Program (NIP) vaccines are administered according to the national schedule and non-NIP vaccines are offered on a voluntary, self-paid basis. The municipal government has implemented supplementary programs to increase immunization coverage and uptake among residents, including provision of varicella and pneumococcal conjugate vaccine (PCV) vaccination services for targeted populations, as well as other supplementary immunization measures.

In China, where public health policies and immunization programs are continuously evolving, understanding the population’s knowledge and attitudes toward non-NIP vaccines is crucial for designing effective interventions. Despite an increasing number of studies on vaccine awareness and acceptance, most research in China has focused on specific vaccines or limited populations, and few have systematically examined the cognitive levels and multifaceted determinants of awareness and acceptance across a broad range of non-NIP vaccines among general urban residents. However, there is still a lack of comprehensive, population-based evidence on how sociodemographic, informational, and psychosocial factors jointly shape non-NIP vaccine perceptions in large urban settings such as Shanghai, which the present study aims to address. This study aims to examine residents’ awareness and influencing factors regarding non-NIP vaccines in a selected urban area, in order to provide evidence-based recommendations for improving vaccination coverage and public health outcomes. Specifically, our study in Shanghai conducted an empirical survey to assess residents’ awareness of and acceptance toward non-NIP vaccines and to identify factors shaping these perceptions. The findings are intended to guide efforts to increase non-NIP vaccine uptake and optimize vaccination services in urban populations.

## 2. Materials and Methods

This population-based cross-sectional survey was conducted in Shanghai, one of China’s largest megacities with a population exceeding 25 million. Shanghai is administratively divided into 16 districts, including both urban and suburban areas, with heterogeneous socioeconomic profiles and varied access to health services. The city has implemented extensive immunization services through CDC and community health centers, providing National Immunization Program (NIP) vaccines free of charge and offering non-NIP vaccines on a voluntary, self-paid basis. The reporting and conduct of this cross-sectional survey followed the STROBE statement, which provides guidance for reporting observational epidemiological studies including sampling strategy, variable definition, and analytic approach.

### 2.1. Study Design and Participants

This population cross-sectional survey was conducted in Shanghai from 20 October to 31 December 2024. We employed a stratified multistage probability sampling design, consistent with standard survey sampling methodology to improve representativeness and precision. First, all 16 administrative districts in Shanghai were stratified into categories (e.g., central urban vs suburban) to form strata, based on geographic and demographic characteristics. We chose five districts based on statistical efficiency and logistical feasibility given our target sample size, consistent with prior surveys, where a similar number of primary sampling units was sufficient to achieve both adequate representation and manageable field operations. The selected five districts included both highly urbanized central areas (such as districts with high population density and advanced health service infrastructure) and more socioeconomically diverse suburban areas, ensuring that the sample captured key variations in demographic, economic, and healthcare access characteristics across Shanghai’s municipal population. Within each stratum, the five districts were selected using simple random sampling, in which each district had an equal probability of being chosen from the list of all eligible districts within that stratum.

Within each selected district, communities (administratively defined neighborhood units equivalent to residents’ committees or residential communities, which serve as standard sampling units in Chinese household and health surveys) were listed, and simple random sampling was used to select four communities per district, ensuring that each eligible community had an equal probability of being chosen. Finally, 35–40 residents per community were randomly recruited to complete a questionnaire. Within each selected community, ‘residents’ refers to adult individuals listed in the community’s official household registry (sampling frame) who met the inclusion criteria, and recruitment was conducted by drawing from this registry using a simple random sampling approach to ensure that every eligible resident in the community had an equal probability of being selected from the sampling frame. The target number of 35–40 residents per community was derived from the overall sample size calculation to ensure sufficient precision for estimating key outcome proportions (e.g., awareness and acceptance levels) while accounting for design effect in a multistage sampling framework [34,35].

Inclusion criteria were adults who had lived in the local area for at least six months in the past year. Exclusion criteria included: (1) individuals with diagnosed severe psychiatric disorders; (2) individuals with serious health conditions; (3) persons with hearing or communication impairments that would prevent effective participation; (4) individuals unable to cooperate for other reasons; (5) aged under 18 years. The required sample size was calculated using the formula *n = Zα*^2^ *× p × q/d*^2^, *q* = 1 − *p*, setting *α* = 0.05 and allowable error *d* = 0.1*p*, and assuming an awareness rate *p* = 35% from prior literature, the estimated sample size was *n* ≈ 742 [36]. This formula is the standard approach for estimating sample size for a population proportion under a simple random sampling framework, and in our stratified multistage probability sampling design it provided a baseline estimate which was then aligned with the sampling strategy to achieve the desired precision [35]. A total of 760 questionnaires were distributed; 753 valid questionnaires were returned (valid response rate = 99.08%). Questionnaires were administered primarily through face-to-face interactions by trained survey staff, the reported figure reflects the proportion of valid responses among the collected surveys (99.08%), and 760 of the 1000 (76.00%, individuals invited to participate) invited individuals completed the survey. The study was conducted in accordance with the Declaration of Helsinki and the protocol was approved by the Institutional Review Board (Ethics Committee) of the Shandong Center for Disease Prevention and Control (protocol code SDJK(K)2024-049-01; date of approval: 9 October 2024).

### 2.2. Questionnaire Development

Initial questionnaire items were derived from policy documents and literature searches in CNKI, Wanfang, and the official website of China’s National Health Commission using keywords such as non-immunization program vaccine, vaccine awareness, and vaccination intention. We also referenced the Knowledge–Attitude–Practice (KAP) model, which conceptualizes how knowledge influences attitudes and, in turn, shapes health behavior, and constructs from the Health Belief Model (HBM), which emphasizes perceived susceptibility, severity, benefits, barriers, and cues to action in health decision-making, to support the conceptual organization of awareness and acceptance constructs in the questionnaire. Reference was made to the National Survey on Procurement and Management of non-National Immunization Program Vaccines (General Public) followed by tailoring of the Residents’ Awareness and Acceptance of non-NIP Vaccines draft questionnaire. Two rounds of expert consultation workshops were held with specialists in immunization planning, vaccine management, health services administration, and statistics to refine item relevance.

Through this expert review process, content validity of the questionnaire was established by ensuring that all items were relevant, clear, and aligned with the study objectives. In addition to expert consultation workshops, the complete questionnaire was pre-tested in a pilot survey with a small sample of community residents representative of the target population. The questionnaire consisted of 49 questions covering sociodemographic characteristics, awareness and acceptance constructs, and potential influencing factors. Completion of the questionnaire took approximately 15 min on average. The purpose of this pre-test was to evaluate item clarity, relevance, response burden, and respondent comprehension. Trained field staff conducted the pilot using the same structured interview mode planned for the main survey. Findings from the pre-test indicated that most items were comprehensible; however, several questions (e.g., those related to recent exposure to vaccine information and perceived affordability categories) were reworded to improve interpretability and reduce ambiguity. Minor modifications focused on simplifying phrasing and clarifying specific response options without changing the conceptual content of the items. The final questionnaire achieved a Cronbach’s α of 0.731, indicating acceptable internal consistency reliability for the set of items used to measure awareness and acceptance constructs (Appendix A).

### 2.3. Variables

Sociodemographic information: gender, age, education level, occupation, self-reported living standard, urban/rural residence type, health status, health insurance status, usual vaccination site, distance to nearest vaccination site, mode of transportation, waiting time, and perceived convenience of access.

All sociodemographic variables were treated as categorical variables. These variables were treated as explanatory variables. Measurement and response options of study variables are presented in Table 1.

Awareness and Acceptance: Respondents’ self-rated level of understanding and degree of endorsement of non-NIP vaccines (five-point Likert scales).

These variables were treated as outcome variables. Measurement and response options of study variables are presented in Table 1.

Additional Factors: (a) Whether a medical professional recommended any non-NIP vaccines; (b) participation in promotional or educational activities about non-NIP vaccines; (c) awareness of any recent negative news or events related to vaccines; (d) knowledge of any free vaccination programs; and (e) perceived match between vaccine price and personal ability to pay (perceived affordability). 

These variables were treated as explanatory variables. Measurement and response options of study variables are presented in Table 1.

The survey did not include direct items soliciting participants’ self-reported reasons for not receiving non-NIP vaccines; instead, we measured a set of explanatory variables that have been shown to be associated with vaccine hesitancy and low uptake, including perceived affordability, recommendation by healthcare personnel, awareness of adverse events, and participation in vaccine education.

### 2.4. Quality Control

Survey administrators received standardized training before data collection. The survey was conducted primarily through face-to-face structured interviews. Trained interviewers administered the questionnaire in person at community health service centers or at respondents’ homes, reading each item verbatim to the respondent and recording responses directly. Face-to-face administration was chosen to ensure clarity of item interpretation and minimize missing data.

The purpose and procedures of the study were explained to all participants, and informed consent was obtained. Before each interview, the survey team described the objectives of the study, assured participants of voluntary participation, and obtained written informed consent. Participants were informed that the survey would take approximately 10 min and that their responses would remain confidential. Interviewers adhered to a standardized protocol for introducing the study, reading each question consistently, and responding to participant queries without leading responses.

Participants were assured that their responses would remain confidential and be used only for research purposes. All questionnaires were anonymized. During field administration, supervisors conducted spot checks to monitor adherence to the protocol, ensure completeness of responses, and address any operational issues. Any questionnaire with missing or ambiguous responses was reviewed with the participant before the end of the interview to minimize item non-response and improve data integrity.

After data collection, responses were entered into a database by a designated clerk and independently verified for accuracy. The database was then checked by another researcher for completeness and logical consistency to ensure data quality. Double data entry procedures were implemented where feasible to further reduce data entry errors. Discrepancies identified during entry and verification were resolved through cross-checking with original paper forms or electronic records.

### 2.5. Statistical Analysis

Data were organized in Excel 2019 and analyzed using SPSS 29.0. During data processing, we examined all variables for missing values; overall, the proportion of missing data was minimal across key variables. Where item non-response occurred, respondents were re-contacted using the contact information provided in the questionnaire to clarify or complete missing responses when feasible; when missing data remained after follow-up, those records were treated as incomplete and excluded from specific analyses (complete-case analysis). Given the stratified multistage probability sampling design, key sociodemographic stratification variables (e.g., district type and community strata) were inspected in preliminary analyses, and models were adjusted for these variables to account for potential design effects on association estimates. To visually summarize the multivariable ordinal logistic regression results, adjusted odds ratios and corresponding 95% confidence intervals were plotted as coefficient plots using R version 4.4.1 with the RStudio platform after multivariable analysis.

Descriptive statistics were first used to summarize participants’ sociodemographic characteristics as well as the distribution of awareness and acceptance levels of non-NIP vaccines, including frequencies and percentages for categorical variables and ordinal outcomes. Because the dependent variables (awareness and acceptance of non-NIP vaccines) were measured on ordinal scales and did not meet the assumptions of normal distribution, non-parametric methods were applied. Descriptive statistics were also presented using frequencies and percentages to summarize the distribution of ordinal and categorical variables. Accordingly, the Kruskal–Wallis H test was used to compare awareness and acceptance scores across different subgroups defined by sociodemographic characteristics and potential influencing factors. This approach is appropriate for comparing ordinal outcomes among multiple independent groups. Variables that showed statistical significance in the Kruskal–Wallis tests were subsequently entered into the multivariable ordinal logistic regression models for further analysis.

Ordinal logistic regression models were applied to identify factors associated with awareness and acceptance levels of non-NIP vaccines. The proportional odds assumption was evaluated using the test of parallel lines, while overall model fit was assessed using the likelihood ratio test. A non-significant result for the parallel lines test supports the proportional odds assumption, and a significant likelihood ratio test indicates that the model with predictors fits the data better than the null model. Multicollinearity among explanatory variables was assessed using variance inflation factors (VIFs), and variables with VIF > 10 were considered to have concerning multicollinearity; no explanatory variables exceeded this threshold. All statistical tests were two-sided, and a *p*-value < 0.05 was considered statistically significant.

## 3. Results

### 3.1. Participant Characteristics

A total of 753 participants were included in the analysis. Of these, 242 (32.1%) were male and 511 (67.9%) were female. Age distribution was largely 21–40 years (47.9%) and 41–60 years (29.1%). The majority had a university education (63.5%), followed by high school level (16.1%). In 2023, most households reported annual incomes in the ranges ¥50,000–100,000 (28.4%) or ¥100,000–500,000 (57.1%). About two-thirds of participants (65.7%) described their household as middle-income, and most respondents (81.8%) lived in urban communities. Details are provided in Table 2.

For context on income levels in the overall Shanghai population, the average income of residents in Shanghai in 2024 was approximately RMB 88,366 according to the 2024 Shanghai Statistical Bulletin on National Economic and Social Development. Urban resident average income was approximately RMB 93,095, while rural resident average income was approximately RMB 45,644. These figures provide a reference for comparing the income distribution of the study sample with the broader economic conditions in Shanghai’s resident population. For context on socioeconomic status in the overall Shanghai population, the per capita disposable income of Shanghai residents in 2024 was approximately RMB 88,366. Urban resident per capita disposable income was approximately RMB 93,095, while rural resident per capita disposable income was approximately RMB 45,644 [37,38,39,40].

### 3.2. Univariate Analysis of Awareness of Non-NIP Vaccines

Respondents self-rated their awareness of non-NIP vaccines as follows: very well aware (15.5%), fairly well aware (18.7%), moderately aware (32.05%), not very aware (27.2%), and completely unaware (6.5%).

Kruskal–Wallis tests (univariate analysis) showed that awareness scores differed significantly across subgroups defined by age, education level, occupation, living standard, self-reported health status, insurance coverage, convenience of access to vaccination sites, recommendation by healthcare provider, and participation in vaccine education (all *p* < 0.05). Detailed subgroup distributions are presented in Table 3.

### 3.3. Univariate Analysis of Acceptance (Endorsement) of Non-NIP Vaccines

Endorsement levels were rated as: strongly accept (20.3%), somewhat accept (24.7%), moderately accept (45.9%), somewhat reject (7.3%), and strongly reject (1.9%). Kruskal–Wallis tests (univariate analysis) indicated that acceptance scores varied significantly by age, education level, occupation, living standard, residence type, self-rated health, usual vaccination site, transportation mode, access convenience, medical recommendation, participation in education, awareness of recent vaccine events, knowledge of free vaccination programs, and perceived affordability of vaccine cost (all *p* < 0.05). Detailed subgroup distributions are presented in Table 4.

### 3.4. Ordinary Multivariable Analysis of Awareness and Acceptance

#### 3.4.1. Awareness Level

**Figure 1:**
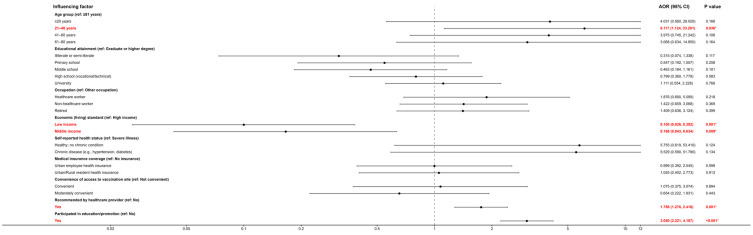
Adjusted Odds Ratios and 95% Confidence Intervals for Predictors of Awareness of non-NIP Vaccines. The red font and the asterisk (*) indicate statistical significance (*p* < 0.05).

Ordinal logistic regression was employed to explore determinants of residents’ awareness of non-NIP vaccines, using self-rated awareness as the dependent variable and variables significant in univariate analyses as covariates. In the model for awareness (likelihood ratio *χ*^2^ = 248.534, *p* < 0.001; parallel lines test *p* = 0.089), significant predictors were age, household living standard, medical recommendation, and educational campaign participation (Table 3). All odds ratios reported in the multivariable ordinal logistic regression analyses are adjusted odds ratios. Compared with respondents aged ≥81, those aged 21–40 had much higher odds of reporting a higher awareness category (OR = 6.117; 95% CI: 1.124–33.281; *p* = 0.036). Household income was strongly associated: individuals from low-income (OR = 0.100; 95% CI: 0.026–0.382; *p* = 0.001) and middle-income (OR = 0.166; 95% CI: 0.043–0.634; *p* = 0.009) households had significantly lower odds of higher awareness than those from high-income households. Receiving a recommendation for non-NIP vaccination significantly increased the odds of higher awareness (OR = 1.756; 95% CI: 1.276–2.416; *p* = 0.001). Participation in non-NIP vaccine education campaigns was associated with much higher awareness (OR = 3.050; 95% CI: 2.221–4.187; *p* < 0.001). Details of the regression coefficients are given in Table 5. The adjusted odds ratios identified in the multivariable ordinal logistic regression models are further illustrated in coefficient plots; see Figure 1 for details. 

#### 3.4.2. Acceptance Level

**Figure 2:**
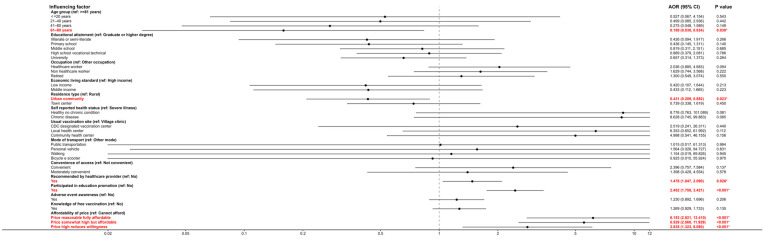
Adjusted Odds Ratios and 95% Confidence Intervals for Predictors of Acceptance of non-NIP Vaccines. The red font and the asterisk (*) indicate statistical significance (*p* < 0.05).

A separate ordinal logistic regression model was fitted with self-rated vaccine acceptance as the response variable, incorporating significant predictors from univariate analysis. In the model for acceptance (likelihood ratio χ^2^ = 177.129, *p* < 0.001; parallel lines test *p* = 0.993), significant predictors included age, residence type, medical recommendation, educational campaign participation, and vaccine affordability (Table 4). All odds ratios reported in the multivariable ordinal logistic regression analyses are adjusted odds ratios. Respondents aged 61–80 had much lower odds of higher acceptance than those ≥81 (OR = 0.159; 95% CI: 0.030–0.834; *p* = 0.030). Urban community dwellers showed lower acceptance than rural residents (OR = 0.431; 95% CI: 0.209–0.892; *p* = 0.023). Receiving a vaccine recommendation significantly increased acceptance (OR = 1.478; 95% CI: 1.047–2.090; *p* = 0.026), as did participating in education campaigns (OR = 2.452; 95% CI: 1.758–3.421; *p* < 0.001). Perceived affordability had the strongest effect: compared to those who said they “cannot afford it and would not consider vaccination,” respondents who reported the price as “reasonable and fully affordable” were over six times more likely to accept vaccination (OR = 6.153; 95% CI: 2.821–13.410; *p* < 0.001). Those who found the price “high but still affordable” (OR = 5.529; 95% CI: 2.560–11.929; *p* < 0.001) or “high and somewhat deterring” (OR = 2.835; 95% CI: 1.323–6.080; *p* < 0.001) also had significantly higher acceptance than the cannot-afford group. Detailed results are shown in Table 6. The adjusted odds ratios identified in the multivariable ordinal logistic regression models are further illustrated in coefficient plots; see Figure 2 for details. 

## 4. Discussion

Based on survey data collected among residents of Shanghai, this study assessed their knowledge and attitudes toward non-National Immunization Program vaccines, and identified factors influencing these perceptions. While this study did not directly elicit participants’ reasons for not vaccinating, associated factors identified suggest economic, informational, and provider-related influences on non-NIP vaccine acceptance. The findings indicate that overall levels of awareness and acceptance of non-NIP vaccines among Shanghai residents remain limited and warrant improvement. Strikingly, a substantial proportion of respondents exhibited high acceptance despite possessing only limited understanding of non-NIP vaccines. Additionally, significant heterogeneity in both knowledge and acceptance was observed, which correlated with individual-level factors, trust mechanisms, and exposure to media campaigns [41,42,43]. These findings align with conclusions from prior studies [44,45,46,47,48].

### 4.1. The Phenomenon of High Acceptance but Low Knowledge of Non-NIP Vaccines Among Residents

This study found that, consistent with prior research on non-National Immunization Program vaccines in China, residents of Shanghai exhibit relatively high acceptance of non-NIP vaccines but lack sufficient specific knowledge [36,45]. In other words, although positive attitudes toward vaccination and a willingness to be vaccinated are widespread, public awareness of critical aspects—including vaccine safety, dosing, vaccination schedules, the need for booster or follow-up doses, side effects, the risk of adverse events, cost burdens, and accessibility—remains limited. This disconnect between positive attitudes and actual knowledge may be a key barrier to improving vaccine uptake [49,50].

The Knowledge–Attitude–Practice (KAP) paradigm conceptualizes vaccination behavior as a process in which knowledge influences attitudes, which in turn shape behavioral intentions and practices. This pattern suggests that positive attitudes toward vaccination may reflect a general confidence in vaccines as valuable preventive health tools, while specific knowledge about non-NIP vaccines represents a distinct cognitive dimension that informs practical decision-making. Although favorable attitudes provide motivational support for immunization, adequate vaccine-specific knowledge is necessary for individuals to interpret nuanced information, assess risk–benefit tradeoffs, and make context-appropriate vaccination decisions.

Consequently, gaps in factual understanding can act as a key barrier that limits the extent to which positive attitudes translate into consistent and informed vaccine uptake in real-world settings. These findings were consistent with a substantial body of research both in China and internationally. For example, in Malawi, Ndasauka found that among 394 adults, those with high vaccine knowledge and positive attitudes were over 8 times more likely to be vaccinated compared to individuals with low knowledge and negative attitudes; combination of knowledge and attitude was more predictive than either alone [23]. In the South Gondar Zone of Ethiopia, a study of 1111 general adults revealed that good knowledge and favorable attitudes toward vaccination were strongly associated with both vaccine acceptance and actual uptake, even after adjusting for sociodemographic variables [28]. A nationwide cross-sectional survey in Malawi found that widespread vaccine misconceptions significantly contributed to low vaccination coverage [29]. Negative perceptions of vaccines are significantly associated with lower rates of vaccine uptake [30,31].

In China, Zhou et al., in their comparative study of local urban, migrant, non-left-behind, and left-behind households in Zhejiang and Henan provinces, observed that local urban families had substantially higher vaccination coverage among children and demonstrated high awareness of basic non-NIP vaccine items such as vaccine types, timing and schedule, and disease prevention categories. However, recognition of vaccine continuity—such as booster doses, additional doses, and long-term immunity—was relatively low. Although these families generally acknowledged the existence and public health value of non-NIP vaccines, knowledge gaps and misunderstandings were widespread when confronting operational concerns or worries about adverse reactions [51].

In Shanghai, a survey of 1691 parents by Wu et al. reported that 69.5% of parents expressed willingness to have their children receive non-NIP vaccines. However, when queried about vaccine safety, the existence of side effects, whether the price is reasonable, and the convenience of vaccination, the proportion of parents with adequate specific knowledge was not particularly high—especially regarding detailed issues such as adverse reactions and safety [36].

In rural regions, Wang et al., through qualitative research, found that about 75% of respondents considered children’s non-NIP vaccination necessary and important. However, most respondents could not name the specific diseases targeted, dosing requirements, schedules, or actual efficacy of these vaccines. Many parents identified vaccines by disease names rather than by vaccine type, lacking awareness of specific details (for example, some did not realize that the EV71 vaccine prevents hand–foot–and–mouth disease). As a result, they often relied on hearsay or incomplete information and had little understanding of the true likelihood of side effects [49].

Finally, a cross-sectional study conducted in Zhejiang Province among guardians of adolescent girls—using the Health Belief Model framework—found that guardian acceptance of the HPV vaccine was high (86.7%). However, knowledge about the recommended target population for HPV vaccination (only 27.91%) and similar details remains comparatively low [50]. Collectively, these findings indicate that in China, public attitudes toward non-NIP vaccines tend to be positive, but critical knowledge gaps persist—a gap that may hinder real-world uptake.

### 4.2. Factors Influencing Awareness

Age, household living standard, recommendation by medical personnel, and participation in vaccine education were identified as critical determinants of awareness. Notably, individuals aged 21–40 years showed markedly higher awareness than those aged ≥81, echoing prior observations that younger adults generally possess stronger health literacy and more proactive information-seeking behaviors [52]. Meanwhile, the age ≥81 cohort showed exceptionally low awareness, underscoring the vulnerability of the oldest age group to being left behind in vaccine-related health communication. Young and middle-aged people have high information acquisition ability and health awareness, and can actively understand vaccine related knowledge through the Internet and other channels [53]. However, it should be noted that the complexity of information dissemination also makes it possible for some young and middle-aged people to mix different sources when receiving information, which is easy to form knowledge misunderstandings, and puts forward higher requirements for the further consolidation of scientific cognition [54].

Economic disparities also played a substantial role: those in low-income and middle-income brackets displayed significantly lower awareness compared with high-income respondents. This aligns with literature showing that socioeconomic status influences access to and comprehension of health information, often limiting exposure in lower socioeconomic status populations [55]. Healthcare providers’ recommendation emerged as a strong predictor of awareness, reinforcing the documented role of professionals in overcoming knowledge barriers through personalized, credible guidance [56,57]. Participation in organized vaccine education and promotion was associated with the highest odds of increased awareness. This suggests that systematic intervention programs can substantially boost vaccine knowledge retention. These patterns highlight the importance of tailored outreach: educational platforms combining medical authority, media literacy, and community-based campaigns could be particularly effective at elevating public understanding—especially among older adults and economically disadvantaged groups [58]. Therefore, future efforts should continue to strengthen vaccine-related health communication centered on healthcare professionals, combining both new media and traditional media channels. Targeted science-popularization campaigns should be implemented to expand the breadth and depth of vaccine knowledge dissemination across different population groups.

### 4.3. Factors Influencing Acceptance

In terms of acceptance, our study identified age, type of residence, recommendation by healthcare personnel for non-NIP vaccine administration, participation in non-NIP vaccine promotional and educational activities, and the alignment of vaccine price with individuals’ payment capacity as the principal determinants influencing willingness to vaccinate.

From a theoretical perspective, the observed determinants of non-NIP vaccine acceptance align with constructs of the Health Belief Model (HBM), a well-established framework in health behavior research that explains preventive health actions such as vaccination. According to HBM, individuals’ decisions to engage in preventive behaviors are influenced by perceptions of both disease risk (susceptibility and severity) and vaccination benefits and barriers, as well as cues to action provided by trusted sources. In the context of vaccine uptake, cues such as recommendation by healthcare providers and participation in educational activities can be viewed as external triggers that enhance perceived benefits and reduce perceived barriers, thereby increasing acceptance. Empirical studies applying HBM to vaccination have consistently identified perceived benefits, perceived barriers, and cues to action as key predictors of vaccination intentions and uptake, underscoring the relevance of these constructs for interpreting factors associated with non-NIP vaccine acceptance observed in the present study.

First, compared with residents aged 81 years and older, those aged 61–80 exhibited a lower level of acceptance toward non-NIP vaccines. This finding suggests that among middle-aged and older adults, vaccine acceptance does not monotonically decline with increasing age—indeed, some very old individuals (≥81 years) might display higher acceptance in practice, potentially due to greater burden of chronic diseases or higher dependence on healthcare services. In contrast, the 61–80 age group, despite being at high risk for chronic illness, may be more susceptible to negative public discourse in information consumption, leading to misconceptions-which corresponds to a tendency toward greater caution or conservatism toward health interventions [59]. Increased doubts among some older people about vaccine safety and necessity may thus contribute to the relatively low acceptance of non-NIP vaccines in this age cohort; accordingly, more targeted communication strategies are needed to deliver tailored vaccine education and outreach efforts [60].

The differences in residential types are also worth paying attention to. The level of recognition of non-immunization vaccines among urban community residents is lower than that among rural residents, which is similar to previous research findings [46]. Urban community residents typically have access to a greater variety of information sources regarding vaccination; however, these sources may be complex or biased, which can lead to lower acceptance of non-National Immunization Program (non-NIP) vaccines [61]. This suggests that, especially in urban areas, it is imperative to strengthen the dissemination of authoritative and evidence-based vaccine information, leveraging both new media and traditional media, and to promptly correct misinformation, in order to provide the public with reliable, accessible information and minimize the interference of erroneous content [62].

Our findings indicate that recommendation of non-NIP vaccines by healthcare personnel significantly increases residents’ acceptance. This underscores that healthcare providers serve a critical guiding role in vaccine decision-making, and their influence should be further strengthened. Prior studies have likewise demonstrated that, as trusted sources of health information, provider recommendations markedly enhance individuals’ vaccine attitudes [63,64]. Especially for non-immunization planned vaccines, where professional endorsement tends to carry greater weight and more readily translates into vaccination behavior, thereby boosting acceptance of non-NIP vaccines [65].

Participation in non-NIP vaccine promotional and educational programs significantly increased residents’ acceptance of such vaccines. Effective dissemination of information enhances acceptance by providing a completer and more systematic framework of vaccine knowledge. Through interactive educational processes, these programs reinforce the public’s risk perception and confidence in non-NIP vaccines, thereby improving overall vaccine acceptance [66]. When doubts about vaccine safety, efficacy, and necessity have substantially increased among older adults and the broader population, targeted training helps address their concerns [67], clarify misconceptions, facilitate effective communication, and build trust—reducing undue concerns about vaccine safety and re-establishing scientific confidence in vaccination within a complex and pluralistic information environment [68].

The higher the alignment between vaccine price and individuals’ payment capacity, the greater the acceptance of non-NIP vaccines. This reflects that economic accessibility remains a major constraint limiting widespread uptake of non-NIP vaccines. Perceived financial burden can significantly suppress vaccination willingness, especially among low-income populations. Therefore, to improve the accessibility and equity of non-NIP vaccines, it is necessary to further optimize vaccine payment and financing mechanisms [69]. On the one hand, using health-economic evaluation outcomes as the basis, cost–effectiveness analyses, cost–benefit assessments, and budget-impact analyses should be conducted to scientifically evaluate the suitability and prioritization of incorporating non-NIP vaccines into local or national immunization programs, and actively promote their inclusion in public payment schemes [70,71]. On the other hand, it is worth exploring the establishment of diversified shared-payment systems, such as government fiscal subsidies, modest coverage via health-insurance accounts, and supplemental coverage from commercial health insurance—to lower the actual out-of-pocket threshold for high-value vaccines, thereby improving public acceptance of non-NIP vaccines [72,73,74].

### 4.4. Limitations

Although this study has a certain degree of representativeness and an adequate sample size—using stratified random sampling and multiple statistical methods to explore residents’ awareness of non-NIP vaccines in Shanghai—it has several limitations that merit consideration: First, cross-sectional design: This was a cross-sectional survey, so causal relationships between variables cannot be determined. For example, although recommendations from healthcare workers and educational interventions were significantly associated with residents’ awareness and acceptance of non-NIP vaccines, it cannot be established that the recommendations or education occurred before the formation of knowledge and attitudes. Reverse causation or confounding by other variables is possible. Second, self-reported data: The survey relied on self-reported questionnaire data, which may introduce recall bias. Participants might overestimate their knowledge due to social desirability (feeling they ought to be informed), or may not accurately recall whether they received vaccine education or recommendations from healthcare provider. Third, sampling limitations: Although stratified random sampling covered multiple communities and districts, selection bias may still be present. Additionally, the sample size calculation was based on a prior study in one district of Shanghai. Future research could expand the sampling frame or increase the sample size to improve representativeness. Finally, although we included a broad set of explanatory variables, this study did not capture certain potentially relevant variables, such as vaccination status, which should be addressed in future research.

### 4.5. Public Health Relevance and Study Strengths

This study’s multimethod analytical approach—starting with Kruskal–Wallis tests to identify potential predictors and followed by multivariable ordinal logistic regression—enabled robust identification of factors independently associated with both awareness and acceptance of non-NIP vaccines. The use of ordinal measures for awareness and acceptance and appropriate statistical models strengthened the validity of effect estimates, while consideration of a broad set of explanatory variables including sociodemographic characteristics, provider recommendation, education participation, and affordability provided a comprehensive view of determinants shaping vaccine perceptions. Although the cross-sectional design limits causal inference and self-reported data may be subject to recall bias, the findings illuminate key behavioral and informational barriers that can be targets for public health action.

The identified associations between knowledge, attitudes, and acceptance suggest clear directions for public health policy. Tailored communication strategies that emphasize evidence-based information on non-NIP vaccine effectiveness and safety, combined with enhanced provider recommendation and structured educational campaigns, could mitigate misconceptions and improve vaccine confidence. Furthermore, aligning financing mechanisms to reduce perceived financial barriers may help translate positive attitudes into actual uptake. These insights can inform efforts to refine China’s immunization framework, particularly in considering how non-NIP vaccines might be better integrated into population health planning to reduce disease burden and promote equitable access.

## 5. Conclusions

This study systematically assessed the levels of knowledge and acceptance of non-NIP vaccines among residents of Shanghai. The findings indicate that overall awareness and acceptance remain below optimal levels, characterized by a pronounced phenomenon of relatively high acceptance but insufficient understanding. These results underscore the need to reinforce the professional recommendation role of healthcare providers and to implement comprehensive educational interventions aimed at improving access to accurate vaccine information, particularly among older adults and low-income populations. Concurrently, efforts to enhance vaccine acceptance should focus on strengthening the dissemination of evidence-based vaccine information, expanding public education initiatives, optimizing vaccine payment mechanisms, and improving affordability. Together, these findings highlight key demand-side barriers relevant to public health practice in China and suggest that exploring diversified financing strategies, such as targeted subsidies or expanded insurance support, may promote more equitable access to high-value non-NIP vaccines. Collectively, these measures could contribute to improving non-NIP vaccine uptake and enhancing the effectiveness and equity of China’s immunization system.

## Figures and Tables

**Table 1:** Measurement and Response Options of Study Variables.

Variable	Response Options
Age group (years)	≤20; 21–40; 41–60; 61–80; ≥81
Gender	Male; Female
Educational attainment	Illiterate or semi-literate; Primary school; Middle school; High school (vocational/technical); University; Graduate or higher degree
Occupation	Healthcare worker; non-healthcare worker; Retired; Other occupation
Economic (living) standard	Low income; Middle income; High income
Residence type	Urban community; Town center; Rural
Self-reported health status	Healthy, no chronic conditions; Chronic disease (e.g., hypertension, diabetes); Severe illness
Medical insurance coverage	Urban employee health insurance; Urban/Rural resident health insurance; No insurance
Usual vaccination site	CDC-designated vaccination center; Local health center; Community health center; Village clinic
Distance to nearest vaccination site	<1 km; 1–3 km; 3–5 km; 5–10 km; >10 km
Mode of transport to vaccination site	Public transportation; Personal vehicle; Walking; Bicycle/e-scooter; Other mode
Waiting time at vaccination site	≤15 min; 15–30 min; 30–60 min; >60 min
Convenience of access to vaccination site	Convenient; Moderately convenient; Not convenient
Awareness and acceptance of non-NIP vaccines	Five-point Likert scales (self-rated understanding and endorsement)
Received recommendation from healthcare provider	Yes; No
Participation in non-NIP vaccine education/promotion	Yes; No
Awareness of recent vaccine-related adverse event reports	Yes; No
Knowledge of free vaccination programmes	Yes; No
Affordability of vaccine price	Price reasonable and fully affordable; Price somewhat high but affordable; Price high and reduces willingness; Cannot afford; Will not consider vaccination

**Table 2:** Sociodemographic characteristics of the study participants.

Characteristic	Category	*n*	%
Gender	Male	242	32.1
	Female	511	67.9
Age group (years)	≤20	16	2.1
	21–40	361	47.9
	41–60	219	29.1
	61–80	151	20.1
	≥81	6	0.8
Educational attainment	Illiterate or semi-literate	10	1.3
	Primary school	37	4.9
	Middle school	78	10.4
	High school (vocational/technical)	121	16.1
	University	478	63.5
	Graduate or above	29	3.9
Occupation	Healthcare worker	176	23.4
	Non-healthcare worker	404	53.7
	Retired	131	17.4
	Other	42	5.6
Annual household income (2023)	<¥20,000	40	5.3
	¥20,000–49,999	45	6
	¥50,000–99,999	214	28.4
	¥100,000–499,999	430	57.1
	≥¥500,000	24	3.2
Self-reported living standard	Low income	249	33.1
	Middle income	495	65.7
	High income	9	1.2
Residence type	Urban community	616	81.8
	Town center	104	13.8
	Rural	33	4.4

**Table 3:** Univariate Analysis of Factors Associated with Residents’ Knowledge Level of non-National Immunization Program (non-NIP) Vaccines.

Influencing Factor	Total Respondents (*n* (%))	Very Well Aware (%)	Fairly Well Aware (%)	Moderately Aware (%)	Not Very Aware (%)	Completely Unaware (%)	Test Statistic	*p*-Value
Age group (years)							54.939	<0.001
≤20	16 (2.12%)	12.50%	12.50%	37.50%	31.25%	6.25%		
21–40	361 (47.94%)	20.22%	22.44%	32.96%	19.94%	4.43%		
41–60	219 (29.08%)	15.07%	17.81%	34.70%	26.03%	6.39%		
61–80	151 (20.05%)	5.96%	12.58%	25.17%	45.70%	10.60%		
≥81	6 (0.80%)	0.00%	0.00%	33.33%	33.33%	33.33%		
Educational attainment							58.945	<0.001
Illiterate or semi-literate	10 (1.33%)	0.00%	20.00%	10.00%	50.00%	20.00%		
Primary school	37 (4.91%)	8.11%	8.11%	29.73%	43.24%	10.81%		
Middle school	78 (10.36%)	10.26%	11.54%	15.38%	46.15%	16.67%		
High school (vocational/technical)	121 (16.07%)	9.92%	14.05%	31.40%	35.54%	9.09%		
University	478 (63.48%)	18.62%	21.34%	35.56%	20.71%	3.77%		
Graduate or higher degree	29 (3.85%)	17.24%	27.59%	31.03%	20.69%	3.45%		
Occupation							59.303	<0.001
Healthcare worker	176 (23.37%)	22.16%	27.84%	33.52%	13.07%	3.41%		
Non-healthcare worker	404 (53.65%)	15.84%	17.57%	34.16%	26.73%	5.69%		
Retired	131 (17.40%)	6.11%	13.74%	21.37%	46.56%	12.21%		
Other occupation	42 (5.58%)	14.29%	7.14%	38.10%	30.95%	9.52%		
Economic (living) standard							28.378	<0.001
Low income	249 (33.07%)	10.44%	13.65%	31.33%	34.94%	9.64%		
Middle income	495 (65.74%)	17.58%	21.41%	32.12%	23.84%	5.05%		
High income	9 (1.20%)	44.44%	11.11%	44.44%	0.00%	0.00%		
Self-reported health status							15.494	<0.001
Healthy; no chronic condition	572 (75.96%)	16.78%	19.58%	34.62%	22.90%	6.12%		
Chronic disease (e.g., hypertension, diabetes)	178 (23.64%)	11.80%	16.29%	23.60%	40.45%	7.87%		
Severe illness	3 (0.40%)	0.00%	0.00%	33.33%	66.67%	0.00%		
Medical insurance coverage							13.526	<0.001
Urban employee health insurance	616 (81.81%)	16.07%	20.62%	32.79%	24.51%	6.01%		
Urban/Rural resident health insurance	120 (15.94%)	14.17%	9.17%	30.00%	37.50%	9.17%		
No insurance	17 (2.26%)	5.88%	17.65%	17.65%	52.94%	5.88%		
Convenience of access to vaccination site							17.582	<0.001
Convenient	599 (79.55%)	17.70%	19.70%	31.39%	26.21%	5.01%		
Moderately convenient	141 (18.73%)	7.09%	14.18%	34.75%	32.62%	11.35%		
Not convenient	13 (1.73%)	7.69%	23.08%	30.77%	15.38%	23.08%		
Received recommendation from healthcare provider							47.8	<0.001
Yes	463 (61.49%)	20.52%	21.38%	32.40%	21.60%	4.10%		
No	290 (38.51%)	7.59%	14.48%	31.38%	36.21%	10.34%		
Participation in non-NIP vaccine education/promotion							100.836	<0.001
Yes	354 (47.01%)	26.27%	24.86%	29.38%	16.10%	3.39%		
No	399 (52.99%)	6.02%	13.28%	34.34%	37.09%	9.27%		

**Table 4:** Univariate Analysis of Factors Associated with Residents’ Acceptance Level of non-National Immunization Program (non-NIP) Vaccines.

Influencing Factor	Total Respondents (*n* (%))	Strongly Accept (%)	Somewhat Accept (%)	Moderately Accept (%)	Somewhat Reject (%)	Strongly Reject (%)	Test Statistic	*p*-Value
Age group							33.804	<0.001
≤20 years	16 (2.12%)	18.75%	18.75%	62.50%	0.00%	0.00%		
21–40 years	361 (47.94%)	26.04%	28.25%	39.06%	5.82%	0.83%		
41–60 years	219 (29.08%)	19.63%	21.46%	46.12%	9.59%	3.20%		
61–80 years	151 (20.05%)	7.28%	21.19%	60.26%	8.61%	2.65%		
≥81 years	6 (0.80%)	16.67%	33.33%	50.00%	0.00%	0.00%		
Educational attainment							20.84	0.001
Illiterate or semi-literate	10 (1.33%)	20.00%	10.00%	40.00%	20.00%	10.00%		
Primary school	37 (4.91%)	5.41%	18.92%	59.46%	10.81%	5.41%		
Middle school	78 (10.36%)	11.54%	23.08%	52.56%	10.26%	2.56%		
High school (vocational/technical)	121 (16.07%)	16.53%	23.97%	52.07%	4.96%	2.48%		
University	478 (63.48%)	22.80%	25.94%	43.31%	6.69%	1.26%		
Graduate or higher degree	29 (3.85%)	34.48%	24.14%	31.03%	10.34%	0.00%		
Occupation							25.037	<0.001
Healthcare worker	176 (23.37%)	28.41%	30.68%	34.09%	5.11%	1.70%		
Non-healthcare worker	404 (53.65%)	20.79%	22.77%	47.28%	7.67%	1.49%		
Retired	131 (17.40%)	8.40%	24.43%	55.73%	9.16%	2.29%		
Other occupation	42 (5.58%)	16.67%	19.05%	52.38%	7.14%	4.76%		
Economic (living) standard							11.574	0.003
Low-income	249 (33.07%)	15.26%	22.49%	49.80%	9.24%	3.21%		
Middle-income	495 (65.74%)	22.02%	26.26%	44.24%	6.26%	1.21%		
High-income	9 (1.20%)	55.56%	0.00%	33.33%	11.11%	0.00%		
Residence type							7.669	0.022
Urban community	616 (81.81%)	18.67%	23.38%	49.03%	6.98%	1.95%		
Town center	104 (13.81%)	25.96%	32.69%	29.81%	9.62%	1.92%		
Rural	33 (4.38%)	30.30%	24.24%	39.39%	6.06%	0.00%		
Self-reported health status							6.844	0.033
Healthy, no chronic conditions	572 (75.96%)	21.68%	25.35%	44.93%	6.47%	1.57%		
Chronic disease (e.g., hypertension, diabetes)	178 (23.64%)	15.73%	22.47%	49.44%	9.55%	2.81%		
Severe illness	3 (0.40%)	0.00%	33.33%	33.33%	33.33%	0.00%		
Usual vaccination site							11.520	0.009
CDC-designated vaccination center	22 (2.92%)	18.18%	9.09%	50.00%	18.18%	4.55%		
Local health center	55 (7.30%)	29.09%	30.91%	34.55%	5.45%	0.00%		
Community health center	673 (89.38%)	19.61%	24.81%	46.66%	6.98%	1.93%		
Village clinic	3 (0.40%)	0.00%	0.00%	66.67%	33.33%	0.00%		
Mode of transport to vaccination site							27.578	<0.001
Public transportation	149 (19.79%)	14.77%	22.15%	54.36%	6.71%	2.01%		
Personal vehicle	264 (35.06%)	29.55%	26.89%	36.36%	5.30%	1.89%		
Walking	139 (18.46%)	15.83%	28.78%	47.48%	6.47%	1.44%		
Bicycle/e-scooter	200 (26.56%)	15.00%	21.00%	51.00%	11.00%	2.00%		
Other mode	1 (0.13%)	0.00%	0.00%	100.00%	0.00%	0.00%		
Convenience of access to vaccination site							18.217	<0.001
Convenient	599 (79.55%)	22.04%	25.88%	44.91%	6.01%	1.17%		
Moderately convenient	141 (18.73%)	12.77%	21.28%	51.06%	10.64%	4.26%		
Not convenient	13 (1.73%)	15.38%	7.69%	38.46%	30.77%	7.69%		
Received recommendation from healthcare provider							188.304	<0.001
Yes	463 (61.49%)	25.92%	26.78%	40.60%	4.97%	1.73%		
No	290 (38.51%)	11.03%	21.38%	54.48%	11.03%	2.07%		
Participation in non-NIP vaccine education/promotion							147.692	<0.001
Yes	354 (47.01%)	33.05%	26.55%	35.03%	3.95%	1.41%		
No	399 (52.99%)	8.77%	23.06%	55.64%	10.28%	2.26%		
Awareness of recent vaccine-related adverse event reports							35.486	<0.001
Yes	258 (34.26%)	33.72%	23.64%	36.05%	5.04%	1.55%		
No	495 (65.74%)	13.13%	25.25%	51.11%	8.48%	2.02%		
Knowledge of free vaccination programmes							31.299	<0.001
Yes	414 (54.98%)	26.81%	25.60%	41.55%	4.59%	1.45%		
No	339 (45.02%)	12.09%	23.60%	51.33%	10.62%	2.36%		
Affordability of vaccine price							45.604	<0.001
Price reasonable and fully affordable	236 (31.34%)	30.93%	23.31%	40.25%	4.24%	1.27%		
Price somewhat high but affordable	281 (37.32%)	19.93%	29.54%	43.06%	7.12%	0.36%		
Price high and reduces willingness	205 (27.22%)	9.27%	21.46%	57.56%	9.27%	2.44%		
Cannot afford; will not consider vaccination	31 (4.12%)	12.90%	12.90%	38.71%	19.35%	16.13%		

**Table 5:** Multivariate Ordinal Logistic Regression Analysis of Factors Influencing Residents’ Knowledge Level of non-National Immunization Program (non-NIP) Vaccines.

Influencing Factor	Regression Coefficient (β)	Standard Error (SE)	Wald Statistic	*p*-Value	Adjusted Odds Ratio (95% CI)	VIF
Awareness of non-NIP vaccines (ref: very well aware)						
completely unaware	−0.998	1.627	0.377	0.539	0.369 (0.015, 8.935)	
not very aware	1.360	1.628	0.697	0.404	3.896 (0.160, 94.822)	
moderately aware	3.050	1.631	3.496	0.062	21.115 (0.863, 516.978)	
fairly well aware	4.308	1.633	6.956	0.008	74.292 (3.022, 1824.388)	
Age group (ref: ≥81 years)						1.879
≤20 years	1.394	1.007	1.917	0.166	4.031 (0.560, 29.020)	
21–40 years	1.811	0.864	4.392	0.036	6.117 (1.124, 33.281)	
41–60 years	1.380	0.855	2.607	0.106	3.975 (0.745, 21.242)	
61–80 years	1.121	0.805	1.940	0.164	3.068 (0.634, 14.850)	
Educational attainment (ref: Graduate or higher degree)						1.924
Illiterate or semi-literate	−1.154	0.737	2.451	0.117	0.315 (0.074, 1.338)	
Primary school	−0.604	0.535	1.278	0.258	0.547 (0.192, 1.557)	
Middle school	−0.771	0.469	2.696	0.101	0.463 (0.184, 1.161)	
High school (vocational/technical)	−0.224	0.408	0.301	0.583	0.799 (0.360, 1.779)	
University	0.105	0.355	0.088	0.766	1.111 (0.554, 2.228)	
Occupation (ref: Other occupation)						1.140
Healthcare worker	0.629	0.510	1.518	0.218	1.876 (0.690, 5.099)	
Non-healthcare worker	0.352	0.392	0.806	0.369	1.422 (0.659, 3.068)	
Retired	0.343	0.406	0.712	0.399	1.409 (0.636, 3.124)	
Economic (living) standard (ref: high income)						1.088
Low income	−2.306	0.686	11.298	0.001	0.100 (0.026, 0.382)	
Middle income	−1.795	0.683	6.906	0.009	0.166 (0.043, 0.634)	
Self-reported health status (ref: Severe illness)						1.313
Healthy; no chronic condition	1.750	1.137	2.368	0.124	5.755 (0.619, 53.410)	
Chronic disease (e.g., hypertension, diabetes)	1.710	1.141	2.245	0.134	5.529 (0.590, 51.780)	
Medical insurance coverage (ref: No insurance)						1.214
Urban employee health insurance	−0.001	0.477	0.001	0.998	0.999 (0.392, 2.545)	
Urban/Rural resident health insurance	0.054	0.493	0.012	0.913	1.055 (0.402, 2.773)	
Convenience of access to vaccination site (ref: Not convenient)						1.052
Convenient	0.072	0.536	0.018	0.894	1.075 (0.375, 3.074)	
Moderately convenient	−0.424	0.552	0.589	0.443	0.654 (0.222, 1.931)	
Recommended non-NIP vaccine by healthcare provider (ref: No)						1.346
Yes	0.563	0.163	11.969	0.001	1.756 (1.276, 2.416)	
Participated in non-NIP vaccine education/promotion (ref: No)						1.312
Yes	1.115	0.162	47.579	<0.001	3.050 (2.221, 4.187)	

**Table 6:** Multivariate Ordinal Logistic Regression Analysis of Factors Influencing Residents’ Acceptance Level of non-National Immunization Program (non-NIP) Vaccines.

Influencing Factor	Regression Coefficient (β)	Standard Error (SE)	Wald Statistic	*p*-Value	Adjusted Odds Ratio (95% CI)	VIF
Acceptance (endorsement) of non-NIP Vaccines (ref: strongly accept)						
strongly reject	−0.315	2.884	0.012	0.913	0.730 (0.003, 208.096)	
somewhat reject	1.509	2.878	0.275	0.600	4.522 (0.016, 1274.106)	
moderately accept	4.572	2.885	2.511	0.113	96.737 (0.339, 27,667.120)	
somewhat accept	6.046	2.888	4.384	0.036	422.420 (1.471, 121,297.320)	
Age group (ref: ≥81 years)						1.946
≤20 years	−0.641	1.053	0.370	0.543	0.527 (0.067, 4.154)	
21–40 years	−0.696	0.904	0.592	0.442	0.499 (0.085, 2.936)	
41–60 years	−1.290	0.894	2.082	0.149	0.275 (0.048, 1.589)	
61–80 years	−1.838	0.845	4.734	0.030	0.159 (0.030, 0.834)	
Educational attainment (ref: Graduate or higher degree)						1.830
Illiterate or semi-literate	−0.854	0.768	1.236	0.266	0.426 (0.094, 1.917)	
Primary school	−0.830	0.562	2.182	0.140	0.436 (0.145, 1.311)	
Middle school	−0.200	0.493	0.165	0.685	0.819 (0.311, 2.151)	
High school (vocational/technical)	−0.118	0.434	0.074	0.786	0.889 (0.379, 2.081)	
University	−0.420	0.376	1.249	0.264	0.657 (0.314, 1.373)	
Occupation (ref: Other occupation)						1.156
Healthcare worker	0.711	0.425	2.800	0.094	2.036 (0.885, 4.683)	
Non-healthcare worker	0.488	0.400	1.489	0.222	1.629 (0.744, 3.568)	
Retired	0.262	0.439	0.356	0.550	1.300 (0.549, 3.074)	
Economic (living) standard (ref: High-income)						1.181
Low-income	−0.868	0.696	1.553	0.213	0.420 (0.107, 1.644)	
Middle-income	−0.838	0.688	1.484	0.223	0.433 (0.112, 1.665)	
Residence type (ref: rural)						1.117
Urban community	−0.841	0.371	5.147	0.023	0.431 (0.209, 0.892)	
Town center	−0.302	0.4	0.570	0.450	0.739 (0.338, 1.619)	
Self-reported health status (ref: Severe illness)						1.326
Healthy, no chronic conditions	2.172	1.247	3.037	0.081	8.776 (0.763, 101.089)	
Chronic disease (e.g., hypertension, diabetes)	2.155	1.249	2.975	0.085	8.628 (0.745, 99.883)	
Usual vaccination site (ref: Village clinic)						1.027
CDC-designated vaccination center	0.924	1.197	0.596	0.440	2.519 (0.241, 26.311)	
Local health center	1.849	1.162	2.533	0.112	6.353 (0.652, 61.992)	
Community health center	1.609	1.134	2.011	0.156	4.998 (0.541, 46.155)	
Mode of transport to vaccination site (ref: Other mode)						1.024
Public transportation	0.015	2.093	0.001	0.994	1.015 (0.017, 61.313)	
Personal vehicle	0.447	2.094	0.046	0.831	1.564 (0.026, 94.727)	
Walking	0.143	2.093	0.005	0.945	1.154 (0.019, 69.826)	
Bicycle/e-scooter	−0.078	2.093	0.001	0.970	0.925 (0.015, 55.924)	
Convenience of access to vaccination site (ref: Not convenient)						1.080
Convenient	0.874	0.588	2.212	0.137	2.396 (0.757, 7.584)	
Moderately convenient	0.335	0.603	0.310	0.578	1.398 (0.429, 4.554)	
Received recommendation from healthcare provider (ref: No)						1.425
Yes	0.391	0.176	4.929	0.026	1.478 (1.047, 2.090)	
Participated in non-NIP education/promotion (ref: No)						1.376
Yes	0.897	0.170	27.918	<0.001	2.452 (1.758, 3.421)	
Awareness of recent vaccine-related adverse event reports (ref: No)						1.215
Yes	0.207	0.164	1.602	0.206	1.230 (0.892, 1.696)	
Knowledge of free vaccination programs (ref: No)						1.223
Yes	0.238	0.159	2.232	0.135	1.269 (0.929, 1.733)	
Affordability of vaccine price (ref: cannot afford)						1.142
Price reasonable; fully affordable	1.817	0.398	20.851	<0.001	6.153 (2.821, 13.410)	
Price somewhat high but affordable	1.710	0.393	18.956	<0.001	5.529 (2.560, 11.929)	
Price high reduces willingness	1.042	0.389	7.181	<0.001	2.835 (1.323, 6.080)	

## Data Availability

The raw data that support the findings of this study are not publicly available due to privacy/ethical restrictions. The data are available from the corresponding author upon reasonable request.

## Abbreviations

The following abbreviations are used in this manuscript:

NIP	National Immunization Program
non-NIP	non-National Immunization Program
CDC	Center for Disease Control/Prevention
OR	odds ratio
CI	confidence interval
SD	standard deviation

## Appendix A. Shanghai Survey Questionnaire on the Current Status of Non-NIP Vaccine Procurement and Use (General Public)

Dear Sir or Madam,

You are invited to participate in a research survey. Before you decide whether to take part, please make sure you understand the purpose of the study and what it entails. Please read the following information carefully. If anything is unclear or if you need more information, ask the research staff on site.

The purpose of this study is to understand the management of non-National Immunization Program (non-NIP) vaccines in China, in order to provide evidence for formulating national immunization strategies. This study is conducted as an online survey. non-NIP vaccines mainly include, but are not limited to, pneumococcal vaccines, meningococcal vaccines, Haemophilus influenzae type b (Hib) vaccine, hand–foot–and–mouth disease (EV71) vaccine, influenza vaccine, HPV vaccine, rotavirus vaccine, varicella (chickenpox) vaccine, pentavalent (five-in-one) combination vaccine, herpes zoster (shingles) vaccine, hepatitis B vaccine, and other common self-paid vaccines. In this survey, you will need to fill in some basic personal information. All data will be kept confidential and will not be disclosed to anyone outside the research team. If results are published, none of your personal information will be revealed. All data will be stored for 10 years. The entire survey takes about 15 min to complete.

Your participation in this study is completely voluntary. If you decide to participate, you will be asked to sign an informed consent form. Even after signing, you are free to withdraw from the study at any time without giving a reason. Withdrawing from the study will not affect your relationship with the research team. —National non-NIP Vaccine Procurement and Use Survey Team

1. Personal Basic Information
1.1. District: ________ District
1.2. Gender: A. Male; B. Female
1.3. Date of birth: ________ Year ____ Month
1.4. Educational attainment:
   A: Illiterate or semi-literate
   B: Primary school
   C: Middle school
   D: High school (including vocational/technical school)
   E: University (college undergraduate)
   F: Graduate or higher degree
1.5. Your occupation:
   A: Agriculture/forestry/animal husbandry/fishery industry worker
   B: Transportation, construction, production or manufacturing industry worker
   C: Enterprise management personnel
   D: Commercial or service industry staff
   E: Government or public institution staff (excluding healthcare workers)
   F: Healthcare worker
   G: Homemaker or self-employed (freelancer)
   H: Retired
   I: Other: ____________
1.6. Total household annual income in 2023:
   A: Below ¥20,000
   B: ¥20,000–50,000
   C: ¥50,000–100,000
   D: ¥100,000–500,000
   E: Above ¥500,000
1.7. Household living standard in your local area:
   A: Low income
   B: Middle income
   C: High income
1.8. Type of residence:
   A: Urban community
   B: Town center
   C: Rural area
1.9. Household members living together with you: (Select all that apply.)
   A: Child(ren) aged 0–6 years
   B: Youth/adolescent(s) aged 7–17 years
   C: Adult(s) aged 18–60 years
   D: Elderly person(s) over 60 years old
   E: No cohabiting family members
1.10. Your health status:
   A: Healthy, no diseases or conditions
   B: Have a chronic disease (e.g., hypertension, diabetes)
   C: Have a serious illness
1.11. Which medical insurance do you have:
   A: Urban employee basic medical insurance
   B: Urban or rural resident medical insurance
   C: None (uninsured)
1.12. In the past year, have you or any family member experienced a major health event (e.g., serious illness, hospitalization)?
   A: Yes
   B: No
1.13. Which vaccination site do you usually visit for vaccinations?
   A: CDC-designated vaccination center
   B: Local health center
   C: Community health center
   D: Village clinic/station
   E: Other: ____________
1.14. Distance from your home to the nearest vaccination site:
   A: <1 km
   B: 1–3 km
   C: 3–5 km
   D: 5–10 km
   E: >10 km
1.15. Mode of transport you typically use to reach the vaccination site:
   A: Public transportation
   B: Personal vehicle (drive yourself)
   C: Walking
   D: Bicycle or e-scooter
   E: Other: ____________
1.16. Your usual waiting time when getting vaccinated:
   A: 15 min or less
   B: 15–30 min
   C: 30–60 min
   D: More than 60 min
1.17. Do you consider it convenient for your household to get to the vaccination site?
   A: Convenient
   B: Moderately convenient
   C: Not convenient
1.18. Contact information (kept strictly confidential):
   Name: ________________ Phone/Email: ________________
2. Overall Awareness of and Factors Influencing non-NIP Vaccines
2.1. How would you describe your level of understanding of non-National Immunization Program (non-NIP) vaccines?
   A: Very well aware (fully understand)
   B: Fairly well aware
   C: Moderately aware
   D: Not very aware
   E: Completely unaware
2.2. What is your level of support for non-NIP vaccines (i.e., do you think they should be provided)?
   A: Not supportive at all
   B: Slightly supportive
   C: Moderately supportive
   D: Quite supportive
   E: Fully supportive
2.3. How satisfied are you with the provision of non-NIP vaccine services?
   A: Completely unsatisfied
   B: Slightly satisfied
   C: Moderately satisfied
   D: Quite satisfied
   E: Completely satisfied
2.4. Has any medical professional ever recommended that you receive a non-NIP vaccine?
   A: Yes
   B: No
2.5. Have you ever participated in any promotional or educational activities about non-NIP vaccines?
   A: Yes
   B: No
2.6. Do you have any preference regarding domestic (Chinese-made) vs. imported vaccines?
   A: No preference
   B: Prefer domestic vaccines
   C: Prefer imported vaccines
2.7. How safe do you believe non-NIP vaccines are?
   A: Very unsafe
   B: Unsafe
   C: Neutral
   D: Safe
   E: Very safe
2.8–2.10. Please indicate your level of agreement with the following statements about vaccine safety. (Use a 1–5 Likert scale, where 1 = “strongly disagree” and 5 = “strongly agree.”)
2.8. I believe that getting vaccinated is safe and the side effects are controllable. (Rate 1–5)
   A: 1 B: 2 C: 3 D: 4 E: 5
2.9. The risk of a serious adverse reaction from vaccination is very low. (Rate 1–5)
   A: 1 B: 2 C: 3 D: 4 E: 5
2.10. Imported vaccines are higher in quality and are worth choosing. (Rate 1–5)
   A: 1 B: 2 C: 3 D: 4 E: 5
2.11. Are you aware of any recent negative news or events related to vaccines?
   A: Yes
   B: No
2.12. In your view, what is the public’s overall level of acceptance of non-NIP vaccines?
   A: Very low
   B: Low
   C: Moderate
   D: High
   E: Very high
2.13. What do you think are the main benefits of vaccination? (Please rank the following in order of importance, 1 = most important)
   A: Preventing disease
   B: Reducing the risk of infection
   C: Lowering treatment costs
   D: Enhancing immunity
2.14. In your locality, have you ever wanted to get a vaccine but found that it was not available (out of stock)?
   A: No
   B: Yes—for example: ________________ (which vaccine?)
2.15. In the past two years, have you gotten any non-NIP vaccine because of any of the following reasons? (Select all that apply.)
   A: Recommendation from a doctor or medical institution
   B: Government or health department publicity campaign
   C: Advice or recommendation from friends/relatives
   D: Media reports or advertisements
   E: Requirement for overseas travel
   F: Requirement of your job or profession
   G: Other reason: ________________
2.16. What do you think are the reasons why you or your family members have not received non-NIP vaccines? (Select all that apply.)
   A: Vaccine cost is too high
   B: Lack of knowledge about the vaccine
   C: Doubts about the vaccine’s effectiveness
   D: Worries about vaccine safety
   E: No perceived need to vaccinate
   F: No time to go for vaccination
   G: Vaccine shortage or unavailability
   H: Other: ________________
2.17–2.21. When deciding whether to get a non-NIP vaccine, how much influence do the following factors have on you? (Likert scale 1–5: 1 = no influence at all, 5 = extreme influence.)
2.17. My family’s or relatives’ opinions influence my vaccination decision. (1–5 rating)
   A: 1 B: 2 C: 3 D: 4 E: 5
2.18. A doctor’s recommendation is very important to me. (1–5 rating)
   A: 1 B: 2 C: 3 D: 4 E: 5
2.19. I tend to prefer choosing vaccines that are cheaper in price. (1–5 rating)
   A: 1 B: 2 C: 3 D: 4 E: 5
2.20. Vaccine safety is the factor I care about the most. (1–5 rating)
   A: 1 B: 2 C: 3 D: 4 E: 5
2.21. I would consider getting vaccines that are widely promoted in my community. (1–5 rating)
   A: 1 B: 2 C: 3 D: 4 E: 5
2.22. When choosing self-paid vaccines, what is the primary factor you consider?
   A: The price of the vaccine
   B: The effectiveness of the vaccine
   C: Potential adverse reactions or side effects
   D: Recommendation from the government or medical institutions
   E: Opinions of family members
2.23. Are you aware of any free vaccination programs (besides NIP vaccines) either locally or in other regions?
   A: Yes
   B: No
2.24. If free vaccination programs (e.g., for HPV, influenza, etc.) were offered in your area, would you choose to get vaccinated (for yourself or your children)?
   A: Yes
   B: No
2.25. If you would still choose not to get vaccinated even when vaccines are free, what concerns are the reasons?
   A: Worries about vaccine safety
   B: Do not feel vaccination is necessary
   C: Other: ________________
2.26. Do you feel that the current prices of vaccines are compatible with your ability to pay?
   A: The price is reasonable and I can fully afford it
   B: The price is somewhat high but I can manage to afford it
   C: The price is rather high and it reduces my willingness to vaccinate
   D: I cannot afford it, so I will not consider getting vaccinated
2.27. Does the current price of vaccines affect your decision to get self-paid (non-NIP) vaccines?
   A: No effect—I think the price is reasonable
   B: Some effect—but I am willing to bear the cost
   C: Yes, the price is high and it affects my decision to vaccinate
   D: Yes, I find the current price unacceptable and will not get self-paid vaccines
2.28. If the price of non-NIP vaccines were lowered, would you be more willing to get vaccinated?
   A: Yes—I would vaccinate if the price were greatly reduced
   B: Yes—I would consider vaccinating if the price were slightly reduced
   C: No—price is not a deciding factor for me
2.29. If the cost of non-NIP vaccines were reduced by 10%, 20%, or 30%, would you consider getting vaccinated?
   A: If costs drop by 10%, I would consider vaccination
   B: If costs drop by 20%, I would consider vaccination
   C: If costs drop by 30%, I would consider vaccination
   D: Even with cost reductions, I would still not consider vaccination
2.30. To ensure safety in vaccination, besides the vaccine cost itself, would you be willing to pay for any of the following additional fees at the time of vaccination?
   (Select all that you would accept paying.)
   A: Registration fee
   B: Necessary medical examination fee before vaccination
   C: Consultation service fee for vaccine-related information before vaccination
   D: Other additional fee: ________________
   E: I would not accept paying any additional fees
2.31. If you are willing to pay the above additional fees, what is the maximum amount you would accept to pay each time?
   A: Less than ¥20
   B: Less than ¥50
   C: Less than ¥70
   D: Less than ¥100
   E: More than ¥100
